# Gutter oil detection for food safety based on multi-feature machine learning and implementation on FPGA with approximate multipliers

**DOI:** 10.7717/peerj-cs.774

**Published:** 2021-11-16

**Authors:** Wei Jiang, Yuhanxiao Ma, Ruiqi Chen

**Affiliations:** 1 School of Mechanical, Electrical and Information Engineering, Wuxi Vocational Institute of Arts & Technology, Wuxi, Jiangsu Province, China; 2 New York University, Gallatin School of Individualized Study, New York, NY, United States of America; 3 VeriMake Innovation Lab, Nanjing Renmian Integrated Circuit Co.,Ltd., Nanjing, Jiangsu Province, China

**Keywords:** Gutter oil detection, Machine learning, FPGA, K-NN, Approximate multiplier

## Abstract

Since consuming gutter oil does great harm to people’s health, the Food Safety Administration has always been seeking for a more effective and timely supervision. As laboratory tests consume much time, and existing field tests have excessive limitations, a more comprehensive method is in great need. This is the first time a study proposes machine learning algorithms for real-time gutter oil detection under multiple feature dimensions. Moreover, it is deployed on FPGA to be low-power and portable for actual use. Firstly, a variety of oil samples are generated by simulating the real detection environment. Next, based on previous studies, sensors are used to collect significant features that help distinguish gutter oil. Then, the acquired features are filtered and compared using a variety of classifiers. The best classification result is obtained by k-NN with an accuracy of 97.18%, and the algorithm is deployed to FPGA with no significant loss of accuracy. Power consumption is further reduced with the approximate multiplier we designed. Finally, the experimental results show that compared with all other platforms, the whole FPGA-based classification process consumes 4.77 µs and the power consumption is 65.62 mW. The dataset, source code and the 3D modeling file are all open-sourced.

## Introduction

Gutter oil is usually obtained by mixing and refining illegally reused cooking oil, waste animal oil from slaughterhouses, and waste vegetable oil (Lu & Wu, 2014). It is reported to bring prominent health issues like diarrhea and vomiting, or even cause long-term diseases like fatty liver, hyperlipidemia and cancer (Wong, 2020). Now, not only commercial restaurants (Li, Cui & Liu, 2016) but also some education-related outsourcing providers, like university canteens, use gutter oil for cooking to reduce operation costs (China Daily, 2017). According to recent news articles, this problem even affects the whole world, and becomes even more severe in the period of COVID-19 (Star, 2020; TheSmartLocal, 2020). Due to its noteworthy adverse impacts, Food Safety Administration, for example, has always been working on effective distinguishment and follow-up supervision of gutter oil (China Daily, 2019). However, past detection methods are mainly based on chemical analysis in the lab (Geng, Liu & Beachy, 2015), and qualitative analysis on the spot (Liu, Liu & Lei, 2020). Lab analysis requires field sampling and purification before detection, which is relatively costly and time-consuming, greatly delaying the enforcement process. A more practical way to achieve detection for law enforcement is field qualitative analysis with sensors. Although the detection time is shortened and the devices are more portable, the performance of it is not quite desirable since they are mostly threshold-based and with only one indicator. The problem is, given the fact that different cooking conditions will affect the physical and chemical properties of different types of oil in a very complex and unpredictable way, selecting a one-size-fits-all threshold in actual detection seems arbitrary and absolute. Moreover, illegal businesses may exploit loopholes of single indicator, using chemicals to adjust single indicator used in past detection methods (such as pH value and peroxide value) into the range of normal cooking oil to avoid legal punishment (Li, Cui & Liu, 2016). Hence, there is an urgent need for a more comprehensive and well-rounded portable detector that could take different properties into account and make judgement based on machine learning approaches, instead of threshold-based approaches.

The most common methods to classify oil varieties or to distinguish gutter oil in the laboratory are spectroscopic and chromatographic methods. Liquid chromatography is one example (Wang et al., 2017). After the oil sample undergoes dissolution in isopropanol and extraction, the linear range of the corresponding long-chain aldehydes is detected in different fluorescence ranges to further perform classification. A similar recognition method is adopted: injecting the oil sample into quartz tube, burning optical fiber into a channel using femtosecond laser so that the oil sample can flow through, and finally observe and record its dynamic optofluidic refractive index to complete detection (Lin et al., 2018). Raman spectroscopy (SERS), as the most representative method of chemical analysis, is widely used in the field of liquid component analysis (Gojani et al., 2019; Guo et al., 2020; Hu et al., 2019). It is difficult to filter the capsaicin component in gutter oil, but this component will have an obvious peak value in SERS test, which can be used to quickly identify (Tian et al., 2018) of gutter oil. Detecting gutter oil is inherently chemical analysis. Traditional experimental methods are more accurate and reliable than computer-aided design (Wong, 2020). However, its disadvantage is that the oil samples to be tested need to be brought back to the laboratory for a complete experimental process before the final result can be obtained, which will lead to poor timeliness of testing, professional requirements for testing personnel, and increased costs. Moreover, although there are discriminatory data like capsaicin (Tian et al., 2018) and conductivity (Hu et al., 2019), these laboratory methods have high experimental requirements (Lu & Wu, 2014) and are only applicable for chemically treated (purified) gutter oil. It means that it is not practical enough to be used in field tests, where oils are adulterated.

The most common way for detection with sensors is to electrize the test oil and measure relevant parameters to detect harmful components in gutter oil (Baranowska et al., 2008). In literature (Wang, 2020), also based on capsaicin component, differential pulse voltammetry was used to analyze the linear response signal of the oil sample. If the signal is within a specific range, the sample will then be classified as gutter oil. Similarly, the test of gutter oil can also be completed by measuring the conductivity of oil samples after water bath heating (Hu Xueyao, 2013). Since this method only detects parameters of a single dimension, the accuracy of test results will be affected by temperature, impurities of the sample to be tested and other factors. At the same time, relevant instruments using such methods usually use microprocessor level development platform to complete the corresponding data processing, such as STM32, ESP32, MSP 430, etc. (Ge et al., 2019), which are limited by power consumption and performance.

**Figure 1:**
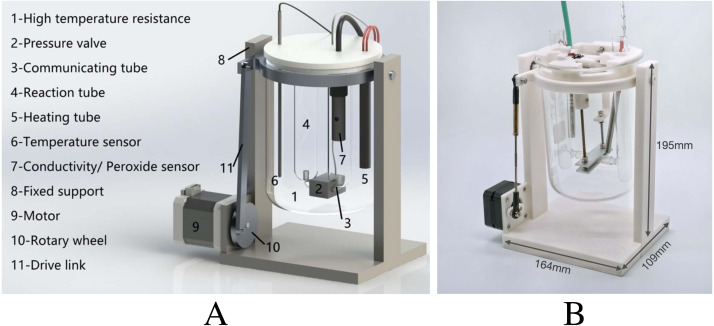
The design of low-power offline device for gutter oil detection. (A) 3D model; (B) 3D printed real product. The STL file for the 3D printed real product is open-sourced.

Aiming at the problems of poor real-time performance, high cost and large error in previous methods, this paper designed a portable low-power real-time detection system for gutter oil based on FPGA, as shown in Fig. 1. In view of the discrimination error caused by a single feature in previous portable detection, we added multiple data dimensions. A machine learning algorithm is introduced to enhance the accuracy of discriminant classification in the case of multi-dimensional data. In addition, all processing operations are completed locally, and the final discriminant classification results are output directly, rather than sending pre-processed data to the high-performance processor to complete the operation. Considering the cost and limited hardware resources of the low-power platform, we further explored the k-Nearest Neighbor accelerator using approximate multiplier to realize the classification and detection of gutter oil. The above functions are realized by FPGA and are compared with other embedded platforms (such as Raspberry Pi). Our main contributions are as follows:

1. A flexible detection system for gutter oil is proposed for the first time based on multi-dimensional features and machine learning.
2. A series of classifiers based on machine learning algorithms are used to comprehensively analyze the multi-dimensional features of gutter oil.
3. An approximate multiplier is proposed based on Intel FPGA platform to save the hardware multiplier and LUT resource consumption in the design process.
4. The classification model k-NN is deployed flexibly on FPGA platform to realize the local classification of oil as well as the detection of gutter oil.
5. The dataset, 3D modeling file for the detector, and the related source code have been made public to help spark progress in the field of food safety testing, and it can also be transferrable to the design of other multi-disciplinary embedded systems.

The rest of the paper is organized as follows: ‘Related work’ reviews relevant literature. ‘Methods’ introduces our research methods in a detailed manner, including our research object, feature acquisition, feature selection and the comparisons between different machine learning classifiers. ‘Hardware implementation of the system’ gives a detailed description of the proposed approximate multiplier. How we implemented the classifier on FPGA and how the system works are explained in ‘The design of approximate multiplier and the deployment results’. In ‘Results and discussion’, we analyzed and evaluated the system in the aspects of performance, resource, and power consumption. Finally, in ‘Conclusions’, we conclude the paper.

## Related work

Just as mentioned in the ‘Introduction’, existing gutter oil detection methods are based on single feature dimension. However, in actual gutter oil detection environments, the mixing of different kinds of oil (Ng et al., 2015), the addition of cooking seasonings (Tokiko Nakayama, 1998) and other external factors make the process even harder. Similar problems exist in the identification of adulterants in edible oils. Instead of single-feature dimension, the researchers proposed multi-feature detection and greatly improved the results.

Giacomelli, Mattea & Ceballos (2006) tested the ability of four principal components, including FA composition, tocopherol levels, CIF (Commission Internationale de l’Eclairage) parameters, and photometric color index in the analysis of edible oils. Data processing algorithm is then implemented on the four-feature original data matrix to classify different vegetable oils. Zhang et al. (2014) proposed a highly effective approach to identify oil authenticity by analyzing the proportions of various fatty acids components, such as caprylic acid, capric acid, lauric acid, and 25 other fatty acids. They further proposed the one-class partial least squares classifier (Zhang et al., 2015) for the identification of peanut oils and the high-precision detection of edible oil adulteration with adulterants even less than 4%.

Thus, based on existing relevant literature, our design draws on both multi-feature detection and their subsequent evaluation process, to the detection of gutter oil. Furthermore, there is hidden correlation under multiple features, so machine learning algorithm can achieve high performance with less feature extraction requirements (Wang et al., 2020b). This is also the reason why machine learning algorithms have been widely used to solve multi-feature problems in various fields.

Liakos et al. (2018) reviewed and concluded that the farming efficiency can be improved by machine learning methods based on multi-sensor’s data. And the works analyzed were categorized in crop management, livestock management, water management, and soil management. The framework is able to produce fast and precise results for simple and compound sentences. Lim et al., (2020) presented a machine learning method to uncover fatty acid patterns discriminative for ten different plant oil types and intra-variability. Moreover, they present a supervised end-to-end learning method that can be generalized to oil composition of any given mixtures. These methods got 50th percentile absolute error between 1.4–1.8% and a 90th percentile error of 4–5.4% for any 3 kinds of oil mix up.

According to the application of the above machine learning algorithms, it can be deduced that the machine learning algorithm brings better results to the problems of multiple data dimension. However, the more features, the more the system needs to compute. Hence most of the tasks are completed in the cloud by general-purpose processors and GPUs with high computing power. It requires a large amount of communication time for the IoT edge terminals (Gubbi et al., 2013), and evidently not suitable for our application scenario. In order to detect gutter oils on the spot and in real time, machine learning algorithms should be deployed to an edge device becomes the central problem to be addressed.

One kind of typical IoT end-point device is the microcontroller unit (MCU). Sakr et al. (2020) investigated machine learning on mainstream microcontrollers and then tested the ability of serial STM MCUs to run machine learning algorithms. Four algorithms were tested, artificial neural network (ANN), decision tree (DT), k-Nearest Neighbors (k-NN), and support vector machine (SVM), with an accuracy above 80% and low power consumption. Ge et al. (2019) implemented a convolutional neural network on FPGA with a Cortex-M3 IP core for image processing. In order to reduce latency, accelerators are designed to be parallel, which further improves its comprehensive performance.

Based on current social context and past research experiences, we first propose a multi-feature machine learning algorithm in the field of gutter oil identification. And then we deployed it to an edge device for on-site detection. Hope it sets the foundation and provides valuable references for future works.

## Methods

This section mainly explains our research object, feature acquisition, feature selection and the chosen machine learning algorithm.

### Research object

The quality of data greatly affects the accuracy results of machine learning algorithms (Xie et al., 2018), so we carefully chose our research object and conducted the following data acquisition (will be explained in detail in ‘Feature acquisition and preliminary analysis’). The research object of this paper is a variety of oils, including gutter oil, chili oil, soybean oil, rapeseed oil, peanut oil and olive oil. With the purpose of simulating real detection environment and different cooking methods, we mixed different types of oils and added different reasoning, adding to the richness of our research object. These oil samples are used for subsequent feature acquisition to form the dataset used in the machine learning algorithm.

**Figure 2:**
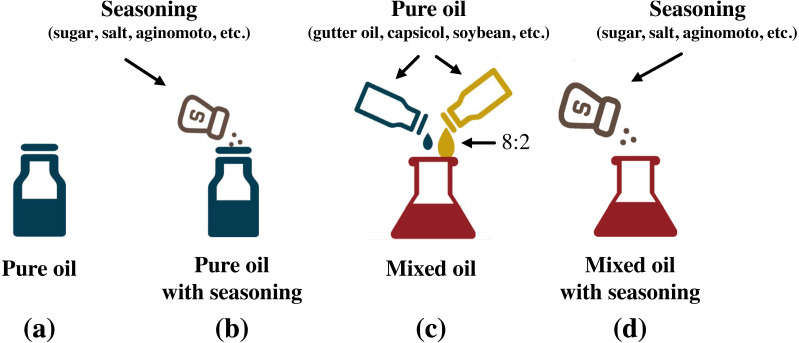
Mixing methods of different oils. (A) Group 1 oils; (B) Group 2 oils; (C) Group 3 oils; (D) Group 4 oils.

The two gutter oil samples are legally acquired from the Food Safety Administration in Fujian Province and Fuzhou Market Supervisory Authority, and they are reported to be the most representative and generalizable ones. Apart from mixing different oils, we also added seasoning that are commonly used in Chinese food, with the quantity they are normally used. They are 5 g sugar, 3 g salt, 2 g monosodium glutamate, 2 g chicken essence, 5 g soy sauce and 10 g vinegar. (Sethi, Chauhan & Anurag, 2017; Wang et al., 2020a; China Daily, 2012; China Daily, 2014; China Daily, 2021) Our study involved in total 3,600 different oils and was made up of 4 groups. There are 15 oils in Group 1, 210 oils in Group 2, 225 oils in Group 3, and 3,150 oils in Group 4. We respectively refer to them as pure oil, pure oil with seasoning, mixed oil, and mixed oil with seasoning, as illustrated in Fig. 2.

In Group 1, as shown in Table 1, there are in total 15 pure oils, 2 gutter oil samples and 13 oil samples chosen from 5 types of cooking oils (chili oil, soybean oil, rapeseed oil, peanut oil and olive oil). 4 common brands of chili oil are selected as our sample because this type of oil contains a large amount of capsaicin, which is also an important indicator of gutter oil detection (Tian et al., 2018). Hence taking into account more chili oil will effectively improve the generalization ability of the whole system. The other oils (soybean oil, rapeseed oil, peanut oil, olive oil) are the most common edible oils used in daily cooking (Shen et al., 2018). The 15 kinds of pure oils are labeled into 6 kinds accordingly.

**Table 1:** Group 1 oils.

Oil type	Label	Size	Total number
pure oil	gutter oil	2	15
	capsicol	4	
	soybean oil	2	
	colza oil	2	
	peanut oil	3	
	olive oil	2	

Group 2 is derived from Group 1 pure oils and there are in total 225 samples as listed in Table 2. We take 25 g of each Group 1 pure oils and respectively add seasonings to them. Based on Chinese cooking habits (Pu et al., 2019), four seasonings are selected from the total of six: 5 g sugar, 3 g salt, 2 g monosodium glutamate, 2 g chicken essence, 5 g soy sauce and 10 g vinegar. Therefore, there are 15 combinations of seasonings to be added to pure oils each time as shown in Formula Eq. (1).

(1) \begin{eqnarray*}{C}_{6}^{4}= \frac{6{!}}{4{!}(6-4){!}} =15\end{eqnarray*}

**Table 2:** Group 2 oils.

Oil type	Label	Size	Total number
pure oil with seasoning	gutter oil	30	225
	capsicol	60	
	soybean oil	30	
	colza oil	30	
	peanut oil	45	
	olive oil	30	

For each oil in Group 1 (15 oils), there will be 15 different combinations of seasonings, so a total of 225 oils (15 × 15) are obtained. And they are labeled in six types just as in Group 1 oils. The reason why we mix seasoning with pure oils is that in field tests, the testing oils are normally used cooking oils collected from food scraps. In this way, various cooking ingredients should be present in testing samples.

Group 3 contains 210 oils, they are obtained by mixing two pure oils (15 × 14), as shown in Table 3. The reason why these mixed oils are taken into the research object is that law enforcement officials reported that some businesses mix edible oils and gutter oils in actual tests to get away with a single-feature detection (Wong, 2020). We set the mixing ratio at 80%:20%, because according to past experiences of authorities, gutter oil is usually mixed with edible oils with more than 30 percent to reduce cost and to avoid detection. In our system, we decided to raise the detection to a higher standard, detecting adulterated oils even when gutter oil is less than 20%. The oil will be labeled as gutter oil as long as gutter oil is mixed into the sample. The other samples are labeled according to the 80% part of it (for example, a mixture of 80% chili oil and 20% soybean oil is labeled as chili oil).

**Table 3:** Group 3 oils.

Oil type	Label	Size	Total number
mixed oil	gutter oil	54	210
	capsicol	48	
	soybean oil	24	
	colza oil	24	
	peanut oil	36	
	olive oil	24	

Group 4 oils are mixed oils with seasoning, and it consists of 3,150 samples as shown in Table 4. It is obtained by adding seasoning just as introduced in Group 2 oils. The same 15 combinations of seasonings are added by the same amount to each 25 g Group 3 mixed oil. Therefore, there are in total 210 × 15 = 3150 different oils. And this group of oils is the closest to the actual detection samples among all four groups, as different oils are usually mixed together with different seasonings.

**Table 4:** Group 4 oils.

Oil type	Label	Size	Total number
mixed oil with seasoning	gutter oil	810	3,150
	capsicol	720	
	soybean oil	360	
	colza oil	360	
	peanut oil	540	
	olive oil	360	

### Feature acquisition and preliminary analysis

The features should be significant enough to reflect the differences between various types of oil. As our ultimate goal is to design a low power consumption, low-cost and portable gutter oil system, the process of feature acquisition should be simple and convenient. For this reason, we consulted a large number of literatures, and based on the portable sensors available in the market, we chose PH0-14 (Hussin, Othman & Tahar, 2019), DJS-1C (Cai et al., 2006), DOM-24 (https://www.atago.net/product/?l=enf=products-dom-top.php), P3-101 (https://i-item.jd.com/10021382704123.html) to collect pH value, peroxide value, electrical conductivity value and refractive index. Considering that data collection in these previous studies was completed under pure oil samples (Turrini, Zunin & Boggia, 2021), we test the usability of the above sensors and conduct preliminary feature analysis with the above four types of feature data on the Group1 research object (Pure Oil).

#### pH values

Gutter oil is usually found in a high percentage of animal fats and bleach, which usually lowers the pH value (Kuuluvainen et al., 2015). PH value sensors (pH 0–14) were used to detect the pH value of 15 pure oil samples selected from 6 different oil types at 25. The pH values of the two gutter oil samples are 3.93 and 8.85, not in the range of normal cooking oils (6.58–7.17). The former one, 3.93, is consistent with the results of previous studies that gutter oil normal has lower pH value. However, the latter 8.85, is obtained by adding excessive alkaline additives. This is probably because illegal traders wanted to lift the pH value but tried too hard (Lu & Wu, 2014).

In addition, temperature has an impact on the pH value of cooking oil (Yang, Qin & Li, 2002), so we need to take it into account. In most previous studies, the upper limit temperature of the test was set at 55 °C 65 °C (Ariaeenejad et al., 2018; Fu et al., 2010). In this paper, instead of acquiring pH value at 25 °C, we took the average and set the upper measuring temperature after heating at 60 °C. According to the data in Table 5, it can be found that the pH value of cooking oil does not change significantly with the increase in temperature, while the change of gutter oil is relatively large as the impurities in it are more sensitive to changes in temperature.

**Table 5:** pH Value of different oils at different temperature.

Oil type	25 °C	60 °C
Gutter oil 1	3.93	3.38
Gutter oil 2	8.85	9.21
Capsicol 1	5.77	5.80
Capsicol 2	6.12	6.16
Capsicol 3	5.98	5.97
Capsicol 4	6.17	6.24
Soybean oil 1	5.94	5.90
Soybean oil 2	5.10	5.12
Colza oil 1	6.99	7.00
Colza oil 2	7.02	7.14
Peanut oil 1	4.98	4.88
Peanut oil 2	4.55	4.50
Peanut oil 3	4.70	4.67
Olive oil 1	5.71	5.73
Olive oil 2	5.51	5.55

#### Conductivity

**Figure 3:**
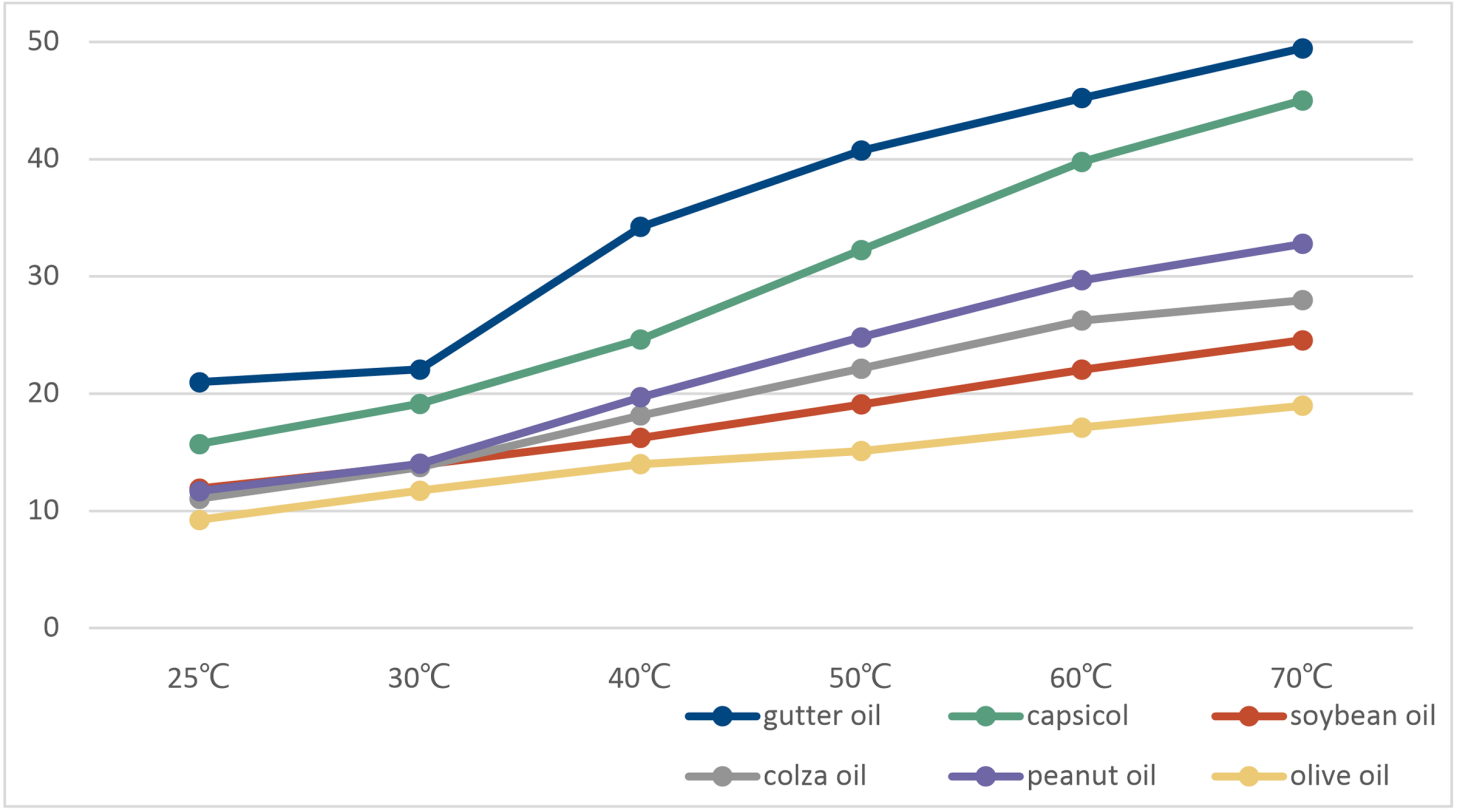
The conductivity of various kinds of oil at different temperature.

Fifteen pure oil samples from Group 1 were heated in a water bath. N-hexane and Deionized water were added to the oil samples at different bath temperatures, and the mixture was continuously oscillated to ensure mixing (Ortega et al., 2019). Then the aqueous phase can be separated and separated through communicators. Conductivity sensor(DJS-1C) was used to measure the conductivity value of its aqueous phase. The average value of oil samples of the same kind was taken, as shown in Fig. 3. It can be found from the data in Fig. 3 that the increase of temperature can effectively reflect the difference between oils through the conductivity value. Therefore, electrical conductivity can be regarded as one of the distinguishing features of gutter oil. Compared with other oil samples, the conductivity value and change rate of gutter oil are generally higher, and the difference increases with the rise of temperature. But after reaching a certain temperature, the change slows down, because the impurities or ions in the oil are limited. On the other hand, according to the latest research (Wang et al., 2019; Okafor & Nwoguh, 2019), there is a hidden relationship between the variation of conductivity at different temperatures and the quality of oil. Therefore, the difference of conductivity at different temperatures can also be used as a distinguishing feature.

 Considering that the water bath heating will consume a lot of time and the change will slow down after the temperature rises, we set the upper limit of the water bath temperature at 60 °C just the same as the temperature we set for the pH data collection. Finally, conductivity value at 25 °C conductivity value at 60 °C and the conductivity value difference between 25 °C and 60 °C are selected to be candidate features.

#### Peroxide value

**Figure 4:**
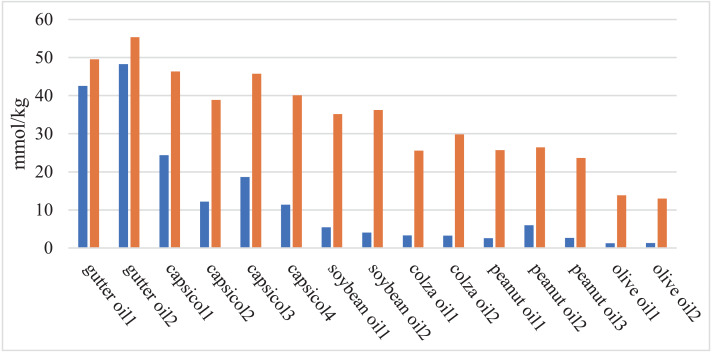
Comparison chart of peroxide value of different oil samples. The blue bar represents the peroxide value at room temperature (25 °C), and the orange bar represents the peroxide value when heated to 60 °C by water bath.

A large amount of peroxide will be produced in cooking oil after heating, so the peroxide value can be used as a distinguishing feature of oil detection (Oishi et al., 1992; ISO 27107:2008, 2008). DOM-24, a special sensor for peroxide value measurement, was used in the experiment. The peroxide value of different types of oils at 25 °C and 60 °C are shown in Fig. 4. It can be seen from the figure that the peroxide value of gutter oil is relatively high, even at 25. The reason is that gutter oil is used repeatedly, the content of decomposable oxides in gutter oil is less than that in cooking oil under same temperature.

And after water bath, the peroxide value of all oil samples increases. While the peroxide value of normal cooking oil shows great differences before and after the heating, gutter oil has no great value changes. Due to this experimental results and the previous conductivity value difference, we proposed to introduce the peroxide value difference between 25 °C and 60 °C.

Finally, Peroxide Value at 25 °C, the peroxide value at 60 °C and the difference between the peroxide value at 25 °C and 60 °C are selected to be the candidate features.

#### Refractive index

Refractive Index is a simplified parameter in the spectrum method and can also be applied to measure the quality of cooking oil (Balestrini et al., 2018). Using P3-101 type refractive index sensor, the refractive index value of oil samples to be tested can be obtained directly. At 25, it was found that the values of 15 pure oil samples to be tested were all between 1.4713 and 1.4768. The value of gutter oil is below 1.4730.

The value of refractive index shows no significant changes with the increase in temperature (Winkler, Proietti & Knoche, 2018), so only the refractive index at 25 °C is considered to be one of the candidate features.

### The selection of specific features

Based on the data obtained by the above mentioned sensors and the preliminary analysis, we listed nine specific candidate features out of four main feature types as shown in Table 6. The data of these nine features obtained as well as their corresponding labels from the dataset of our experiment.

**Table 6:** The candidate features.

Feature types	Specific features
pH value	pH value at 25 °C pH value at 60 °C
Conductivity value	conductivity value at 25 °C conductivity value at 60 °C, conductivity value difference Between 25 °C and 60 °C
Peroxide value	peroxide value at 25 °C peroxide value at 60 °C, peroxide value difference between 25 °C and 60 °C
Refractive index	refractive index at 25 °C

Through the above operation and preliminary analysis, we finally selected the pH value at 25 °C, the peroxide value at 25 °C, conductivity value at 25 °C, the conductivity value at 60 °C, the difference between 60 °C and 25 °C, the peroxide value between 60 °C and 25 °C, and the refractive index at 25 °C as the candidate features as shown in Table 6. In particular, the peroxide value difference between 25 °C and 60 °C is a feature inspired by conductivity value difference.

**Figure 5:**
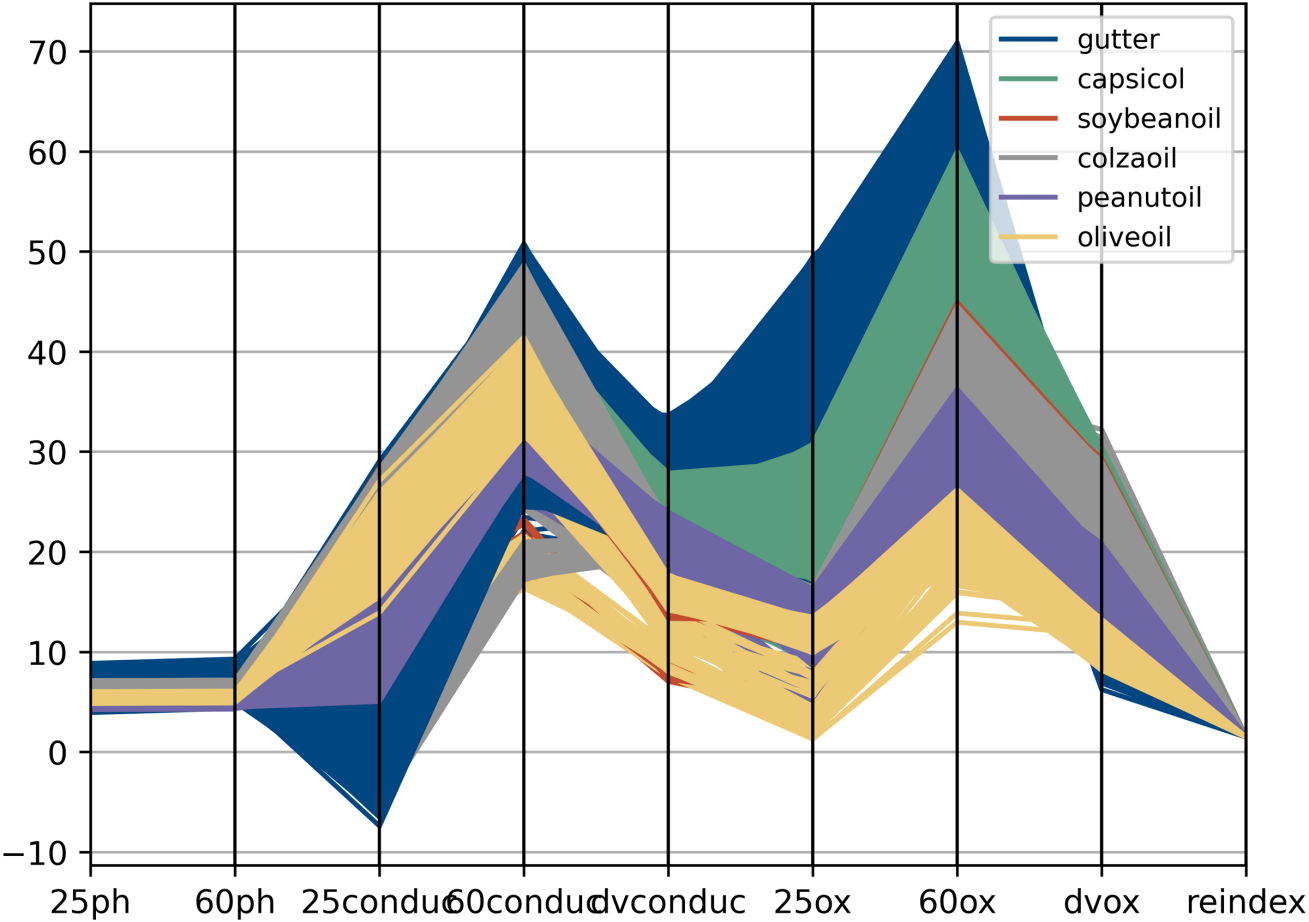
The parallel coordinates plot of the feature dataset. “25ph” refers to the pH value at 25 °C, “60conduc” refers to the conductivity value at 60 °C, “dvconduc” refers to conductivity value difference between 25 °C and 60 °C, “25ox” refers to the peroxide value at 25 °C, “dvox” refers to the peroxide value difference between 25 °C and 60 °C, and “reindex” refers to the refractive index at 25 °C.

The nine-feature data is visualized through a Parallel Coordinates Plot (Inselberg, 1985) as shown in Fig. 5. A parallel Coordinates plot can be used to represent the independent effect of each feature on target prediction. The specific analysis is based on two principles: 1. Whether the broken lines of the same color are concentrated. If the broken lines of the same color are concentrated in a certain feature and there is a certain space between different colors, it indicates that this feature is of great help to predict the label category. 2. If lines are disordered and colors are mixed on a feature dimension, it is more likely that this attribute is of no value for tag category determination.

Since our ultimate goal is to make a low power and low-cost gutter oil detection system, the algorithm should not be too complicated. Therefore, five groups of popular and simple supervised classifiers, including ANN, DT, k-NN, RF and SVM are selected to further evaluate each feature of 3600 oil samples. On the PC side, based on Python, each feature is respectively trained by five classifiers with the scikitlearn machine learning library. Algorithm-specific Configuration Parameters are set by default. For the dataset, we divided the training set and testing set by the ratio of 6:4. And k-fold cross validation was performed with the default cv value (3).

**Figure 6:**
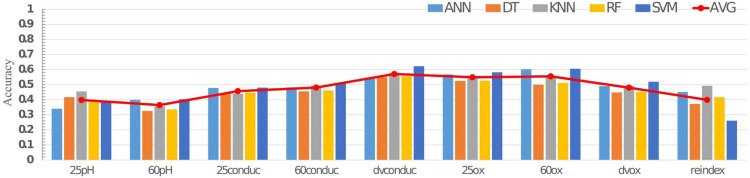
Comparison chart of accuracy of different classifiers. “25 ph” refer to the pH value at 25 °C, “60 pH” refer to the pH value at 60 °C, “60conduc” refer to the conductivity value at 60 °C, “dvconduc” refer to the conductivity value difference between 25 °C and 60 °C, “25 ox” refer to the peroxide value at 25 °C, “60 ox” refer to the peroxide value at 60 °C, “dvox” to the refer peroxide value difference between 25 °C and 60 °C, and “reindex” refer to the refractive index.

Figure 6 shows the accuracy results of each feature under different classifiers. The red line indicates the level of average accuracy of the nine candidate features. Although machine learning algorithm cannot achieve satisfactory results, the classification accuracy of all features is less than 70% . Therefore, the multi-dimensional feature is put forward as the input for the classification and discrimination of gutter oil. Considering that the ultimate goal is a low-power and low-cost gutter oil detection system, limiting the number of input features can reduce the system’s computational burden, thus achieving the savings of operation time and power consumption.

In order to seek a balance between accuracy and input dimensions, we gradually increased the number of input features in the classifier and tested the performance of the eight sets of features. The features are added to the classifier based on their accuracy under single feature dimension, and we recorded each accuracy result obtained in the operation. The results are shown in Table 7 from row 1 to row 8. According to the data in the table, it can be found that when the feature dimension increases, the classification accuracy increases accordingly. And it’s noteworthy that when there are four input features (row 3), dvconduc+60ox+25ox+60conduc, adding new features no longer has an edge in increasing accuracy.

**Table 7:** The accuracy results of different sets of features on different models.

Feature	ANN	DT	k-NN	RF	SVM	AVE
dvcon+ 60ox	0.8275	0.7806	0.8331	0.8267	0.8418	08219
dvcon+ 60ox+ 25ox	0.9483	0.9587	0.9571	0.9626	0.9571	0.9568
dvcon+ 60ox+ 25ox+ 60con	0.9420	0.9750	0.9729	0.9674	0.9634	0.9641
dvcon+ 60ox+ 25ox+ 60con+ dvox	0.9515	0.9777	0.9849	0.9682	0.9658	0.9696
dvcon+ 60ox+ 25ox+ 60con+ dvox+ 25conduc	0.9404	0.9769	0.9809	0.9682	0.9674	0.9668
dvcon+ 60ox+ 25ox+ 60con+ dvox+ 25con+ reindex	0.9531	0.9777	0.9817	0.9682	0.9658	0.9693
dvcon+ 60ox+ 25ox+ 60con+ dvox+ 25con+ reindex+ 25pH	0.9369	0.9777	0.9905	0.9690	0.9626	0.9673
dvcon+ 60ox+ 25ox+ 60con+ dvox+ 25con+ reindex+ 25pH+ 60pH	0.9348	0.9769	0.9928	0.9706	0.9603	0.9671
25pH+ 25con+ 25ox+ reindex	0.8839	0.8943	0.9205	0.8649	0.8482	0.8824

In addition, considering that the heating process takes time in real detection environment, we additionally conducted a multi-feature input classification training for all the features under 25 °C (25pH, 25conduc, 25ox, reindex), to see if the model can achieve a satisfactory result even without heating. However, as shown in Table 7 row 9, the four features obtained at room temperature is not good enough. Its accuracy is 5.24% lower than that of another four-feature input dvconduc+60ox+25ox+60conduc (row 3).

**Figure 7:**
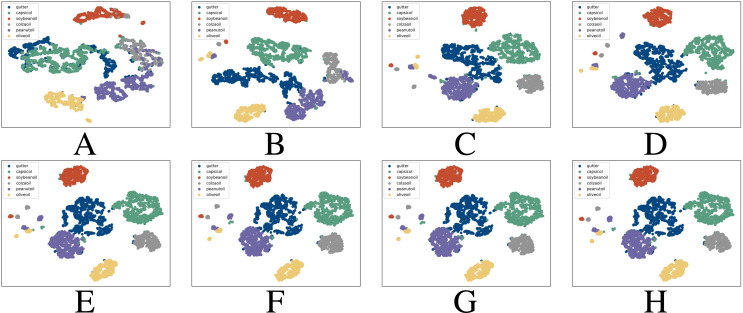
T-Distributed Stochastic Neighbor Embedding (T-SNE) for different sets of features. (A) Single-feature test, (B) two-feature test, (C) three-feature test, (D) four-feature test, (E) five-feature test, (F) six-feature test, (G) seven-feature test, (H) eight-feature test.

We further used T-Distributed Stochastic Neighbor Embedding (T-SNE) to measure the clustering result of different sets of features as in Fig. 7. T-SNE is a nonlinear technology that preserves the neighborhood relationship between data (Maaten, 2014), it reduces the dimension of the multi-dimensional features and can better visualize the results. It can be proven that when the feature dimension is 4 or more, the clustering result is already considerable. Further increasing the feature dimension will not bring significantly better results. Therefore, we finally choose to adopt dvconduc+60ox+25ox+60conduc, and we believe that they are more than sufficient to ensure the correctness of the classification and will not put too much pressure on the resource and power consumption.

Our goal is to design a low-power and portable device, therefore we need to make the most use of power and area by adopting the most effective features. Moreover, to save data acquisition and processing time, we finally chose four features out of the nine specific features as the input of our classifier, including conductivity value difference between 25 °C and 60 °C, peroxide value at 25 °C, peroxide value at 60 °C, and peroxide value difference between 25 °C and 60 °C.

### Model selection

This paper proposes a multi-feature input machine algorithm to classify gutter oil based on four features: conductivity difference between 60 °C and 25 °C, peroxide value at 25 °C, peroxide value at 60 °C, peroxide value difference between 25 °C and 60 °C. This section explores the performance between classifier ANN, DT, k-NN, RF, and SVM to determine the final deployed classifier. Accuracy is usually an important index to evaluate the classification performance, but there will be some deviations when dealing with unbalanced data sets (Le, 2019). In this paper, other evaluation indexes are also introduced to jointly serve as the evaluation of classifiers. Table 8 shows relevant parameters used to evaluate measures based on confusion matrix statistics.

**Table 8:** Relevant parameters of confusion matrix.

Type	Gutter oil	Capsicol	Soybeanoil	Colzaoil	Peanutoil	Oliveoil
TP	TP_1_	TP_2_	TP_3_	TP_4_	TP_5_	TP_6_
FN	FN_1_	FN_2_	FN_3_	FN_4_	FN_5_	FN_6_
FP	FP_1_	FP_2_	FP_3_	FP_4_	FP_5_	FP_6_

For the calculation of three basic evaluation parameters, including Precision, Recall and F1-score (Le et al., 2021), we introduced the weight parameter w according to the proportion of samples to reduce the operation error of the unbalanced dataset. The corresponding operation is as shown in Eqs. (2)–(4).

(2) \begin{eqnarray*}\text{Precision}=\sum _{i=1}^{6} \left( {w}_{i}\times \frac{T{P}_{i}}{T{P}_{i}+F{P}_{i}} \right) \end{eqnarray*}

(3) \begin{eqnarray*}\text{Recall}=\sum _{i=1}^{6} \left( {w}_{i}\times \frac{T{P}_{i}}{T{P}_{i}+F{N}_{i}} \right) \end{eqnarray*}

(4) \begin{eqnarray*}\text{F1Score}= \frac{2\times \text{Precision}\times \text{Recall}}{\text{Precision}+\text{Recall}} \end{eqnarray*}

Kappa is an important metric, especially in terms of unbalanced data sets (Ul Haq Tahir et al., 2019). It measures the result of the classifier from the numerical value. Theoretically, its value range is [ − 1, 1], and the paper rescale to [0, 1]. The greater the Kappa value, the better the classification performance of the model. We assume that all the predicted numbers are *N*, then Kappa Definition is given in Eq. (5):

(5) \begin{eqnarray*}\text{Kappa}= \frac{{P}_{0}-{P}_{e}}{1-{P}_{e}} \end{eqnarray*}

where

(6) \begin{eqnarray*}{P}_{0}= \frac{\sum _{i=1}^{6}T{P}_{i}}{N} \end{eqnarray*}

(7) \begin{eqnarray*}{P}_{e}= \frac{\sum _{i=1}^{6} \left[ \left( T{P}_{i}+F{P}_{i} \right) \times \left( T{P}_{i}+F{N}_{i} \right) \right] }{N\times N} \end{eqnarray*}

Hamming Loss can also be used to evaluate the performance of multi-classification models, and its value represents the proportion of misclassification tags (Park & Read, 2019). This is a loss function, so the optimal value is zero. The number of predicted samples is denoting *N*, and the number of labels is denoting *M* (6 in this paper), then hamming-loss definition is given in Eq. (8)

(8) \begin{eqnarray*}\text{Hammingloss}(y,\hat {y})= \frac{1}{N\times M} \sum _{i=1}^{N}\sum _{j=1}^{M}{XOR}\nolimits \left( {y}_{i,j},{\hat {y}}_{l,j} \right) \end{eqnarray*}

*y* and $\hat {y}$ are respectively actual class label and predicted class label.

In the case of 3,600 oil samples, the training set and testing set are divided by a ratio of 6:4, and 5 different classifiers are tested based on the above evaluation indicators. The numerical results of relevant indicators on the final statistical test set are shown in Table 9. Although RF has the highest accuracy rate, k-NN has a better performance in unbalanced data when combined with other assessment indicators. Therefore, k-NN is used as the classification model for final deployment.

**Table 9:** Difference classifiers performance.

Evaluation metrics	ANN	DT	k-NN	RF	SVM
Accuracy	0.9420	0.9750	0.9729	0.9674	0.9634
Precision	0.8695	0.9713	0.9741	0.9706	0.9669
Recall	0.8657	0.9706	0.9730	0.9674	0.9634
F1-score	0.8664	0.9708	0.9731	0.9678	0.9639
Kappa	0.8349	0.9638	0.9668	0.9600	0.9551
Hamming Loss	0.1343	0.0294	0.0270	0.0326	0.0366

**Figure 8:**
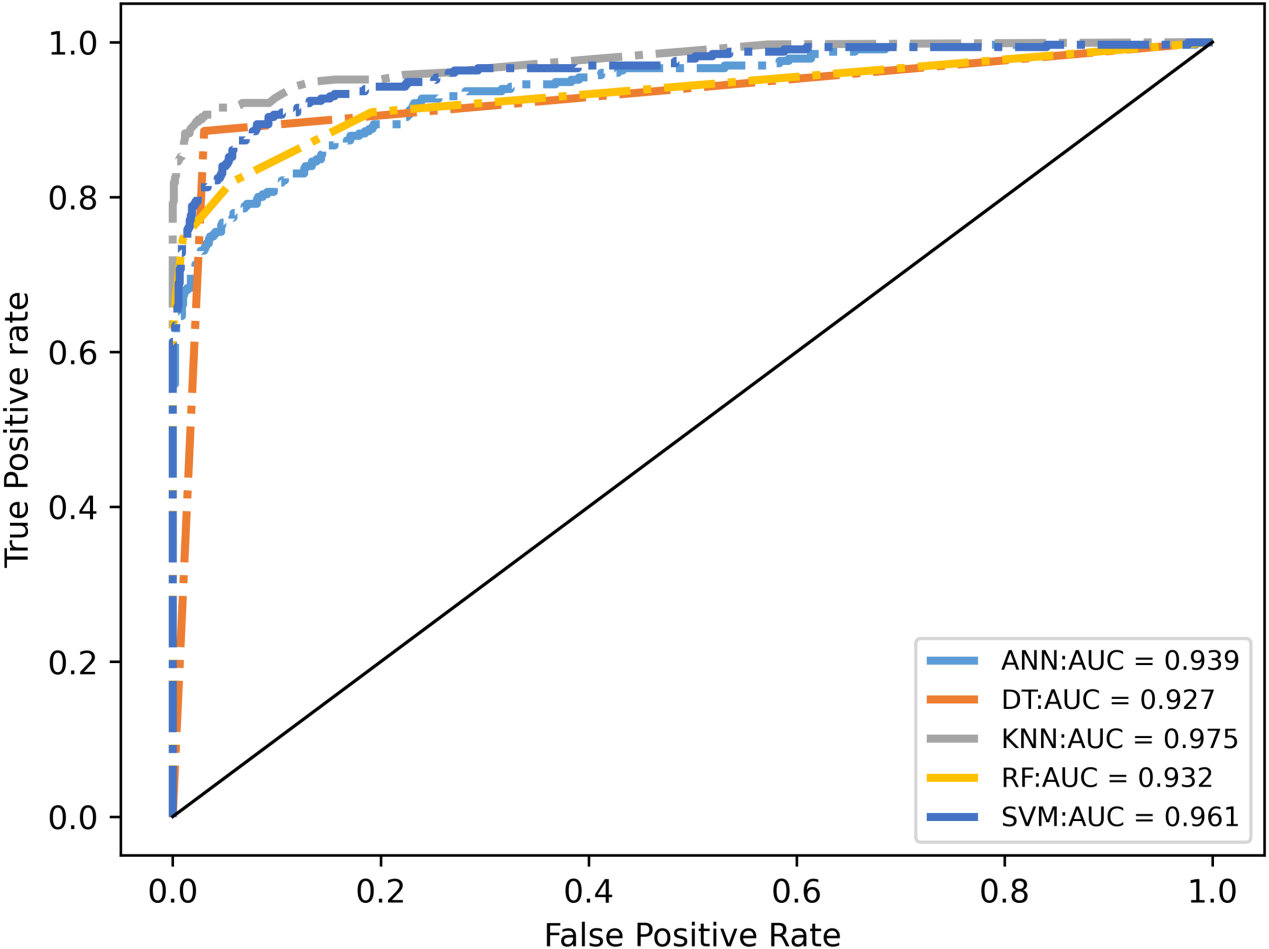
Receiver operating characteristic curve for different machine learning classifiers.

In addition, in order to better evaluate the performance of the following classifiers, the Receiver Operating Characteristics (ROC) and Area Under Curve (AUC) are used as another two indexes, as shown in Fig. 8 (Le et al., 2020). As k-NN, again, demonstrated its strength, it is selected to be the classifier model for the final deployment.

## Hardware Implementation of the System

### Hardware Deployment for Overall Functionality

**Figure 9:**
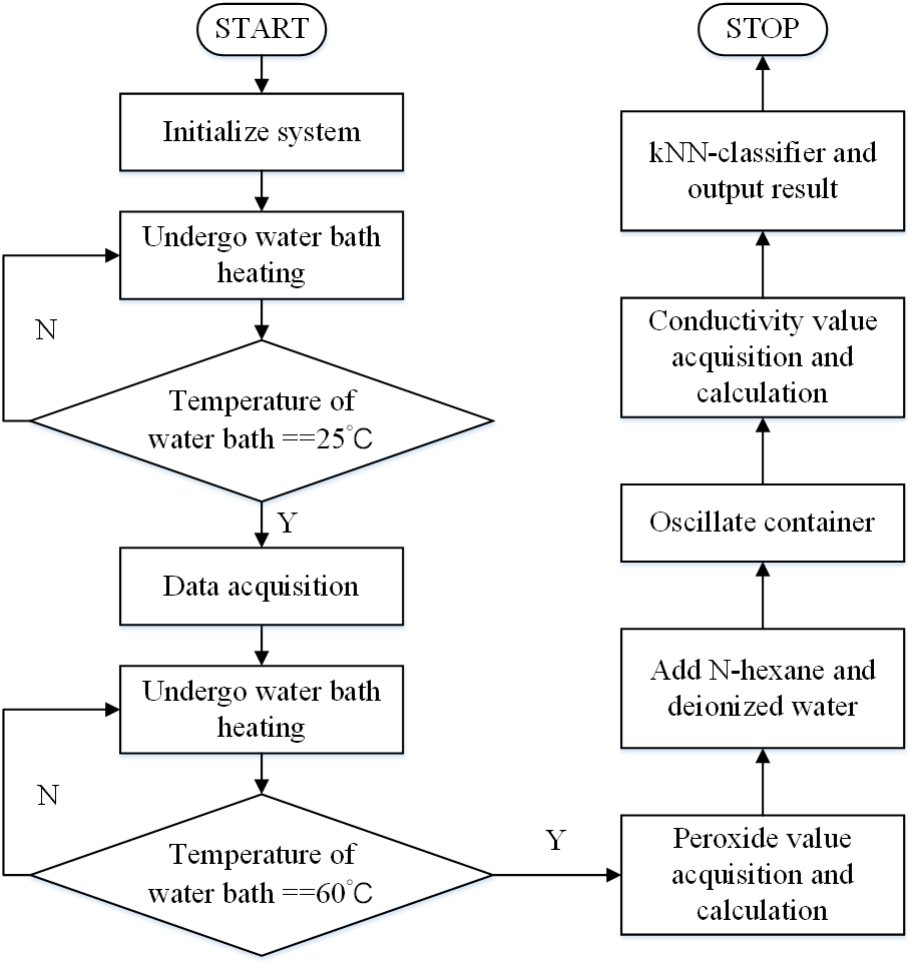
Flowchart of the detection process.

After the selection of features and the comparison of models, specific functional design can be carried out based on relevant results. The whole system completes the data acquisition and classification operation based on the device shown in Fig. 1. The specific process is shown in Fig. 9. Before the operation of the system, the high temperature resistant container 1 shall be filled with pure water to provide a water bath environment, and the oil sample to be tested shall be added to the reaction tube 4. After that, the system shall be started. Firstly, the water bath is heated by electric heating bar 5, while the temperature sensor 6 monitors the water bath temperature in real time. When the water bath temperature reaches 25 °C, relevant sensor 7 (the specific model is described in ‘Feature acquisition and preliminary analysis’) is used to complete the collection and cache of the peroxide value, and electrical conductivity value under 25 °C into THE RAM of FPGA. After that, the water bath was heated to 60 °C. At this point, the peroxide value under 60 °C was collected through sensor 7, and the difference between the peroxide values before and after was calculated into THE RAM of FPGA. At the same temperature, after the peroxide value was collected and calculated, N-hexane and deionized water were added to reaction tube 4. As described in ‘Feature acquisition and preliminary analysis’, the container needs to be oscillated to achieve full mixing. Here, the whole container is oscillated by means of the stepper motor 9 and the corresponding transmission mechanical structure 10, 11. After the oscillation, open the pressure valve 2 and complete the acquisition of water phase through communicator 3. The 60 °C conductivity was measured using the conductivity sensor, and the difference between the front and rear conductivity was calculated. The peroxide value at 25 °C, peroxide value difference, the conductivity value at 60 °C, and the conductivity difference are fed into the k-NN module for oil classification.

 The above process of hardware control, data acquisition and data calculation are all completed in FPGA. Hardware control can be realized by outputting corresponding signals through IO pins, while sensor data acquisition is all realized by ADC. The implementation of the core machine learning algorithm is described in detail here.

### FPGA Deployment of k-NN

**Figure 10:**
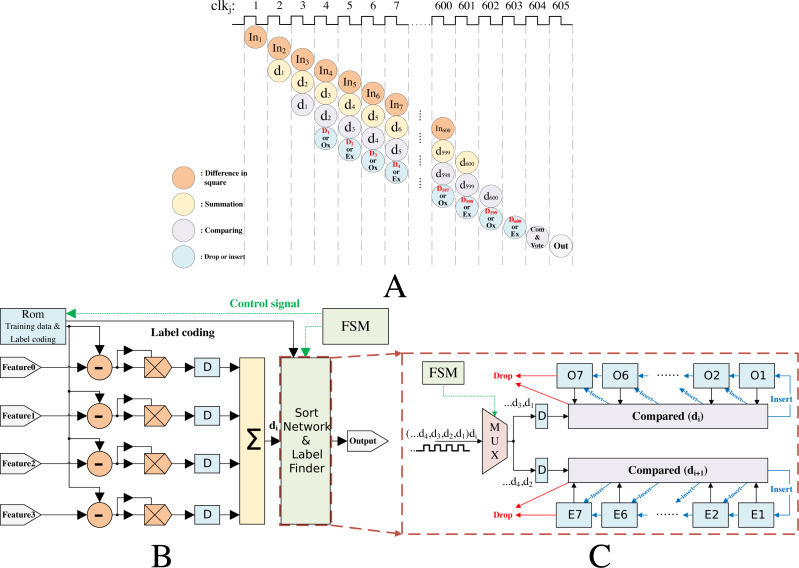
k-NN algorithm implementation. (A) Sequence diagram for the whole operation. In1, In2, and so on refer to the square value. d1, d2 and so on refer to the Euclidian distance. (B) k-NN algorithm architecture for gutter oil detection; (C) architecture for sort network & label finder.

In order to realize the low power k-NN classifier, we made a balance between parallel extensibility and pipeline to reduce the complex operation process as much as possible. Data is allocated and scheduled through Finite State Machine (FSM) to maximize the reuse of computing modules (Attaran et al., 2018). As shown in Fig. 10, the FPGA implementation is explained in detail as follows.

Euclidean distance is selected as the distance function:

(9) \begin{eqnarray*}d=\sum _{i=1}^{4}{ \left( {x}_{i}-{y}_{i} \right) }^{2}\end{eqnarray*}

There are 600 training samples and labels in the ROM block. As shown in Fig. 10B, it scheduled up to four parallel multipliers. Hence, there were two clock cycles to calculate the Euclidian distance (*d*_*i*_) that from input features and training samples. Next, the *d*_*i*_ was inputted into the sort network and label finder module (SNLF) that was showed in Fig. 10C. The mux, comparator, and cache registers constitute the SNLF. The mux was used to control *d*_*i*_ transmit in different clock cycles. The comparator was used to compare with new *d*_*i*_ and storing dis in cache registers. There were 14 cache registers (Ox and Ex) used for storing *d*_*i*_. Specifically, Ox registers were used to store the 7 smallest dis in ‘odd’ clock cycles. Ex registers were used to store the 7 smallest dis in ‘even’ clock cycles. The ping-pong cache was used to rank the *d*_*i*_ in different clock cycles for the SNLF, as shown in Fig. 9A. From those dis, 7 could be identified to be the smallest. Initially we set the register’s value to the maximum. The *d*_*i*_ value compared with each Ox’s value when clock cycle was jth (*j* = 3, 5, 7, …) period. It would be dropped, if *d*_*i*_ value was bigger than ever Ox’s values in next period. Otherwise, *d*_*i*_ value was inserted into Ox and the biggest value in 7 Ox’s would be dropped. Similarly, Ex’s values were updated in (*j* + 1)th (*j* + 1 = 4, 6, 8…) period. The cycles were repeated until all of 600 training samples had been calculated with input features. Finally, comparing all the 14 registers and getting the smallest 7 value from them. The result was voted from the smallest 7 value of registers.

## The design of Approximate Multiplier and the Deployment Results

Among all kinds of operations involved in the deployment of k-NN classifier to FPGA, multiplication operation is the most core. Most FPGAs achieve multiplication by calling IP core or DSP module (Perri et al., 2020). However, there are no related modules on some low-power and low-cost FPGA (Ullah, Nguyen & Kumar, 2020). Therefore, combined with the low power consumption and low-cost design requirements in this paper, an approximate multiplier is proposed according to the device features of Intel FPGA. The above classifier can be provided to complete the corresponding multiplication operation, and at the same time, the resource consumption caused by EDA’s direct multiplication Module Instance can be reduced. This section describes the design and deployment results of this approximate multiplier.

### The design of approximate multiplier

In this paper, the approximate multiplier is realized by recoding some results in the operation process. During the multiplication operation, the multiplier can be split, and the corresponding partial results can be divided into partial product and advance digit, and then the whole multiplication operation process can be completed by adding (Ullah et al., 2018). Because of the split of multiplier in the process of operation, some results can be encoded to enumerate the corresponding results, which is very suitable for LUT (look-up table) structure.

**Figure 11:**
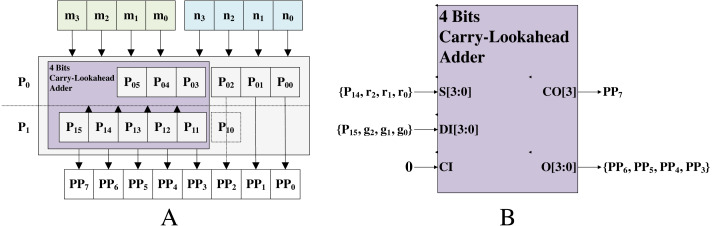
4 × 4 approximate multiplier implementation. (A) The architecture of 4 × 4 approximate multiplier; (B) the architecture of 4 bits carry-lookahead adder.

The specific introduction of this approximate multiplier design process is chosen 4 × 4 approximate multiplier as an example. The 4 × 4 approximate multiplier consists of 4-input LUT, 5-input LUT, and 6-input LUT. The 4-input LUT is a preliminary resource of Intel FPGA. The 5-input LUT is composed of two 4-input LUTs and the 6-input LUT consists of four 4-input LUTs. As shown in Fig. 11A, the *p*_0_ was obtained by *n*_1_*n*_0_ × *m*_1_*m*_0_, and the *p*_1_ was obtained by *n*_3_*n*_2_ × *m*_3_*m*_2_. The *p*_1_ from shifting 2 bits and *p*_0_ were added to produce part of productions (PPs). PPs were summed up to yield the result. Specifically, *p*_0*i*_ (*i* = 0, …, 5) from PPs of *p*_0_, *p*_1*j*_(*j* = 0, …, 5) from PPs of *p*_1_, and *pp*_*k*_(*k* = 0, …, 7) from PPs of 4 × 4 approximate multiplier. All PPs were performed by LUT with enumeration of all possible permutations (initial value), shown in Table 10. The *pp*_0_ and *pp*_1_ were immediately given by *p*_00_ and *pp*_1_. The *pp*_2_ from initial value without carry. The remaining PPs were performed by 4 bits carry-lookahead adder as shown in Fig. 11B. The *r*_*i*_ and *g*_*i*_ (*i* = 0, …, 2) from initial value.

**Table 10:** Initial value and configuration for 4 × 4 approximate multiplier based on LUT.

LUT	Input **I**_5_**I**_4_**I**_3_**I**_2_**I**_1_**I**_0_	Init value (HEX)	Ouput
Lut4_0_	*χχ* 1 1 m_0_ n_0_	8000	P_00_
Lut4_1_	*χχ* m_1_ m_0_ n_1_ n_0_	6aco	P_01_
Lut5_0_	*χ* m_1_ m_0_ n_2_ n_1_ n_0_	b4ccf000	P_02_
Lut6_0_	m_2_ m_1_ n_3_ n_2_ n_1_ n_0_	c738f0f0ff000000	P_03_
Lut6_1_	m_2_ m_1_ n_3_ n_2_ n_1_ n_0_	07c0ff0000000000	P_04_
Lut6_2_	m_2_ m_1_ n_3_ n_2_ n_1_ n_0_	f800000000000000	P_05_
Lut4_2_	*χχ* m_3_ m_2_ n_1_ n_0_	6aco	P_11_
Lut5_1_	*χ* m_3_ m_2_ n_2_ n_1_ n_0_	b4ccf000	P_12_
Lut6_3_	m_3_ m_2_ n_3_ n_2_ n_1_ n_0_	c738f0f0ff000000	P_13_
Lut6_4_	m_3_ m_2_ n_3_ n_2_ n_1_ n_0_	07c0ff0000000000	P_14_
Lut6_5_	m_3_ m_2_ n_3_ n_2_ n_1_ n_0_	f800000000000000	P_15_
Lut5_2_	*χ* P_11_ P_03_ m_2_ n_0_ P_02_	ffeaea00	g_0_
Lut5_3_	*χ* P_11_ P_03_ m_2_ n_0_ P_02_	007f7f80	r_0_
Lut4_3_	*χχ* 1 1 P_12_ P_04_	8000	g_1_
Lut4_4_	*χχ* 1 1 P_12_ P_04_	6000	r_1_
Lut4_5_	*χχ* 1 1 P_13_ P_05_	8000	g_2_
Lut4_6_	*χχ* 1 1 P_13_ P_05_	6000	r_2_
Lut4_7_	*χχ* 1 P_02_ m_2_ n_0_	7800	pp_2_

**Figure 12:**
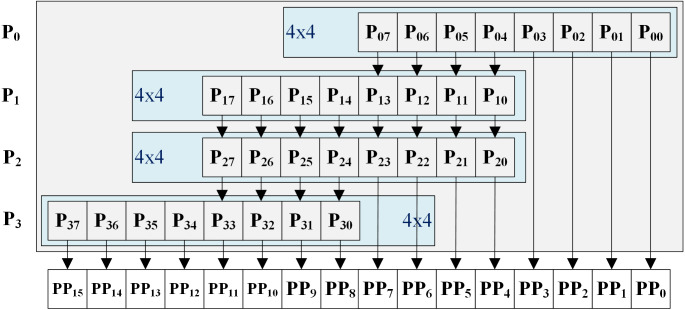
8 × 8 approximate multiplier implementation.

An 8 × 8 approximate multiplier was obtained by four 4 × 4 approximate multipliers as shown in Fig. 12. The PPs of 4 bits (0 to 3) from the first 4 × 4 approximate multiplier (P0) immediately, and the PPs of 4 bits (12 to 15) from the fourth 4 × 4 approximate multiplier (P3) immediately. The remaining PPs were performed by summing all 4 × 4 approximate multipliers without carry. The addition operation implemented on LUT with initial value. Finally, we implement the k-NN with 8 × 8 approximate multiplier.

### The deployment result of approximate multiplier

Using Verilog hardware description language and primitive, an 8 × 8 approximate multiplier was deployed to the Intel FPGA Cyclone 10LP 10CL006ZU256I8G devices which is targeted on Intel Evaluation Kit. The results of the deployment are compared with the relevant studies, as shown in Table 11. The approximate multiplier proposed in this paper is a design based on the balance of area, performance and power consumption. Therefore, although it is not the optimal design in the single dimension of area, performance and power consumption, the proposed approximate multiplier has the optimal performance in the comprehensive index PDP.

**Table 11:** Approximate multiplier performance.

Design	LUTs	Delay (ns)	Power (mW)	PDP (pJ)
Proposed	109	4.72	0.90	4.248
LE-based IP	154	3.70	1.40	5.180
Perri et al. (2020)	116	4.15	1.11	4.607
Venkatachalam & Ko (2017)	101	5.11	0.86	4.395
Griffith (2020)	120	5.00	/	/

**Figure 13:**
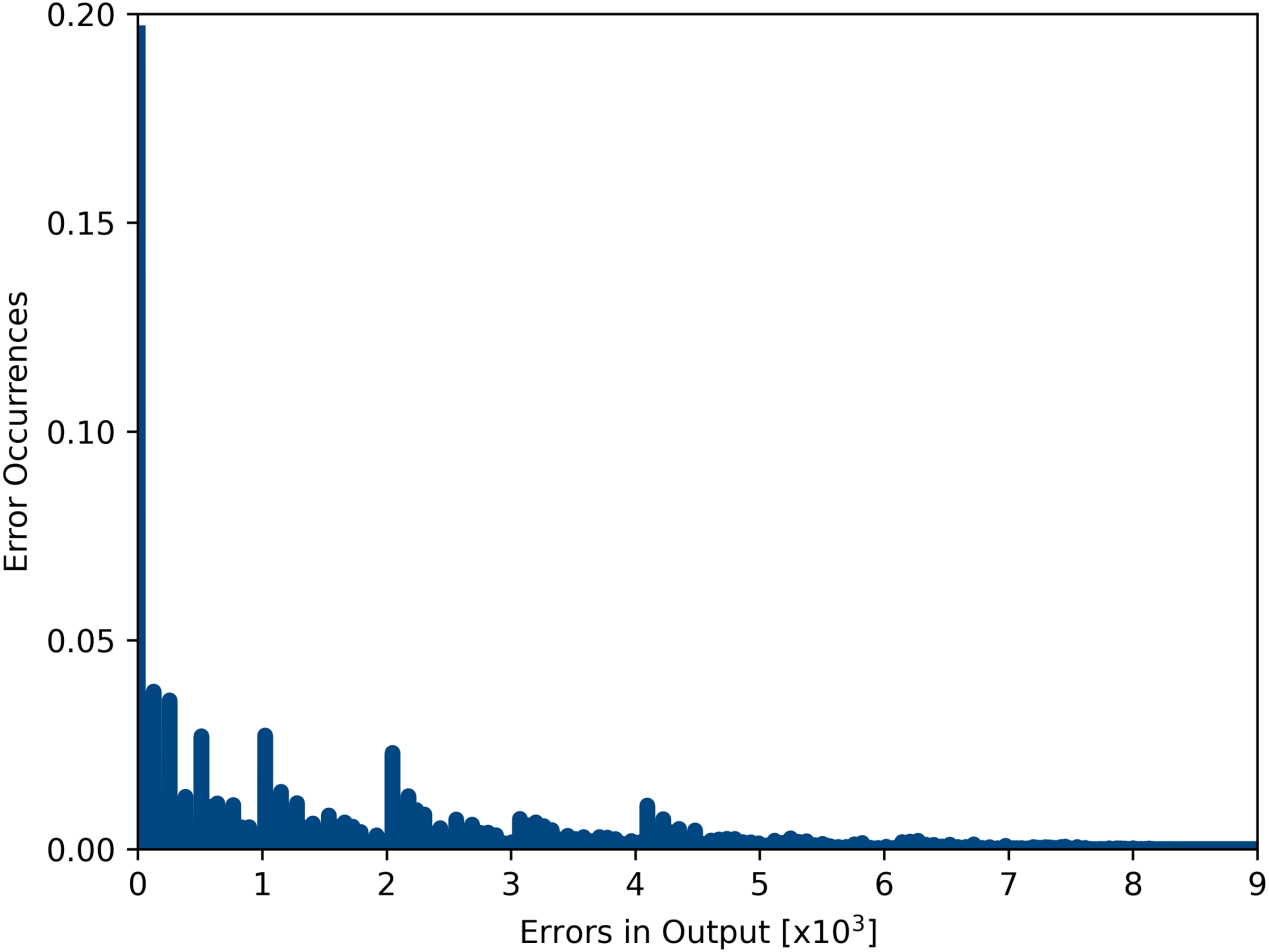
Proportion of error effects of approximate multiplier.

Figure 13 shows the Proportion of Error effects that is a well-adopted quality metric (Rehman et al., 2016) proposed. It can be found that the design of approximate multiplier proposed in this paper controls the errors within the finite size range and frequency.

## Results and Discussion

### Results and analysis

The Intel Evaluation Kit with Intel Cyclone 10LP FPGA as the core serves as a portable platform for low-power gutter oil detection. A driver for external sensors and a k-NN machine learning kernel for feature classification were developed using Verilog. The whole design is based on the design idea of highly parallel and highly pipelined, and an approximate multiplier is designed for devices to further reduce power consumption and resource consumption. Table 12 summarizes the results of the proposed gutter oil detection system conducted by k-NN using an 8 × 8 approximate multiplier on Cyclone 10LP FPGA. And for reference and comparison, a column of LE-based IP performance results is added. While the accuracy of using the original LE-based IP is 97.29%, the classification accuracy after deploying approximate multiplier is 97.18%. Moreover, the entire k-NN design saves approximately 18.7% LUT and reduces 28.90% power consumption. It proves that the design and introduction of approximate multiplier further strengthen the system to be low-cost and low-power.

**Table 12:** Resources used by gutter oil detection system.

Resource & Performance	Consumption	
	Approximate	LE-based IP
LUTs	1,049	1,291
Registers	374	418
Memory (Kb)	19.20	19.20
DSP (Embedded Multiplier)	0	0
Max Freq (MHz)	200	220
Latency (µs)	4.775	3.982
Power (mW)	65.62	92.29

In order to further evaluate the performance of the proposed portable and low-power waste oil detection system, several common embedded development platforms (Rockchip RK3399 Pro, Jetson Nano, Raspberry Pi 3B+, STM32L496) were selected for comparison. Rockchip RK3399 Pro uses Cortex-A72 as its processor core and 2.0 GHz as its main frequency. It is packed with a special NPU module for machine learning. Jetson Nano is cortex-A57 as the core, the main frequency is 1.43 ghz, with a 128-core NVIDIA Maxwell based GPU. The raspberry comes with a 1.2 ghz Cortex-A53 core. STM32L496 is the most common terminal of the Internet of Things, with Cortex-M4 as the core and 80MHz working frequency. On Raspberry Pi and STM32L496, we used serial C language to perform k-NN-based gutter oil detection (Hussin, Othman & Tahar, 2019). In order to perform parallel processing on the GPU, we used PyCuda (Calabrese et al., 2020) on the Jetson Nano platform to achieve parallel processing of k-NN (100 threads processing 600 training data). On the Rockchip platform, the RK-NN-Toolkit tool is used to realize k-NN parallel processing (Lan et al., 2020). Table 13 shows the comparison results for all platforms.

**Table 13:** Proposed gutter oil detection system performance.

Platform	Processor	Clock (MHz)	Lantency (µs)	Power (mW)	PLP
STM32L496(baseline)	Cortex-M4	80	4000	28.38	113520
Raspberry Pi 3B+	Cortex-A53	1200	8.971	1480	13277.08
Jetson Nano	Cortex-A57 with Maxwell-based GPU	1430	0.342	2300	786.60
Rockchip RK3399 Pro	Cortex-A72 with NPU	2000	0.362	2870	1038.94
Our	Cyclone 10LP FPGA	200	4.775	65.62	313.34

**Figure 14:**
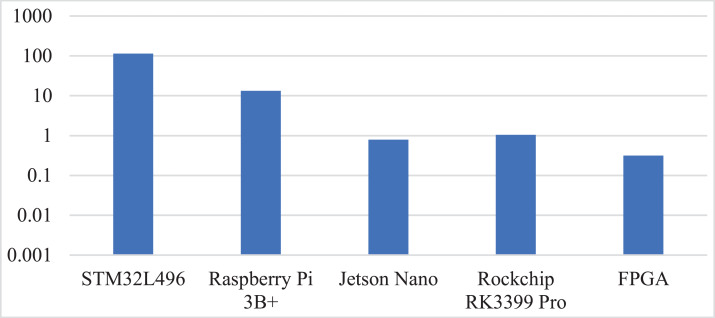
Comparison of power latency production (PLP) for gutter oil detection.

In terms of computing performance, Jetson Nano is the best among all platforms due to the GPU’s high frequency multithreaded parallel processing capability. But in a portable scenario, power and cost become more important: Jetson Nano sells for $129, while Cyclone 10LP FPGA costs just $49. The power latency production (PLP) comparison between all platforms is provided in Fig. 14, which can effectively evaluate the comprehensive performance of the platform (A Kandhalu, 2010). It can be found that THE PLP of FPGA implementation is greatly reduced compared with other platforms. To sum up, FPGA solution, with the best PLP, provides programmability and low development cost, is the best choice for portable and low-power gutter oil detection.

## Discussion and further work

In this paper, a low-power portable detection system for gutter oil is proposed, which uses multi-feature k-NN algorithm with an approximate multiplier deployment on FPGA. It can complete oil classification much more accurately than traditional single dimension detectors. In actual environment for oil detection, laboratory detection methods require the oils to be purified beforehand, which makes the supervision work significantly lagged behind and brings unnecessary economic costs.

Moreover, the multi-feature k-NN algorithm proposed in this paper can effectively extract the hidden associations between different features and results, and the final accuracy reaches 97.18%. Finally, the entire system is deployed on FPGA using approximate multiplier, and it brings the best overall performance compared to other popular embedded platform.

Although the proposed approach and deployment have excellent performance, there is still room for further improvement. While the oil samples we used in this paper were mixed and doped in order to simulate the real detection scene, increasing the samples of gutter oil can help us further train the classifier. Apart from increasing test samples, doping gutter oil with ratios lower than 20% would improve our exploration into the effectiveness of different features and may boost the detection results. However, due to many restrictions beyond our control during the time when the study was conducted, we would add relevant experiments and results in future works.

## Conclusions

Gutter oil used in the catering industry has significant adverse impacts on people’s health and even the society as a whole. Food Safety Administration achieves supervision by sampling and testing oils used by local businesses. However, as gutter oil can be blended with other complex chemicals, existing threshold-based single feature detection could not satisfy the demanding actual detection environment where various interferences may be brought in. In this paper, a portable and low-power gutter oil detection system is designed using machine learning algorithms for the first time to our knowledge. And with our original method that has multi features taken into account, it is much more comprehensive and accurate than past portable detectors.

Based on relevant features including peroxide value and electrical conductivity that have been experimentally proven to be effective, the machine learning classifiers are compared and k-NN was selected for this task. Test results show that all the relevant evaluation parameters perform well under complex detection environment, and the accuracy rate of oil sample classification is up to 97.18%, much higher than previous studies.

Finally, relevant designs are deployed on FPGA, and the core k-NN design achieves a balance between parallel scalability and pipeline to meet the demand of low power consumption. An approximate multiplier is designed for the core multiplication operation, which further reduces the power and resource consumption of the system. While the accuracy of classification model is reduced by only 0.5%, the introduction of approximate multiplier saves 18.7% of resource consumption and 28.9% power consumption. This paper also compares and discusses various embedded IoT terminals to evaluate our design in the aspects of power consumption and performance that offset each other. Experimental results show that the performance of FPGA proposed in this paper is close to two times of Cortex-A53, and PLP improves 2.5 times compared with embedded GPU platform. Under this condition, the circuit resource consumption is only 1,049LUT, and precious hardware multiplication resources are not used because of the introduction of approximate multiplier, so it can fully meet the design requirements of low-power portable gutter oil detection.

## Additional Information and Declarations

### Data Availability

The following information was supplied regarding data availability: Data and code are available at GitHub:
https://github.com/verimake-team/Gutteroildetector.
